# Cancer-associated fibroblasts drive colorectal cancer cell progression through exosomal miR-20a-5p-mediated targeting of PTEN and stimulating interleukin-6 production

**DOI:** 10.1186/s12885-024-12190-0

**Published:** 2024-04-01

**Authors:** Mahsa Ghofrani-Shahpar, Katayoon Pakravan, Ehsan Razmara, Faezeh Amooie, Mojdeh Mahmoudian, Masoumeh Heshmati, Sadegh Babashah

**Affiliations:** 1 Department of Cellular and Molecular Biology, Faculty of Advanced Sciences and Technology, Tehran Medical Sciences, Islamic Azad University, Tehran, Iran; 2 Department of Molecular Genetics, Faculty of Biological Sciences, Tarbiat Modares University, Tehran, Iran; 3 Department of Medical Genetics, School of Medical Sciences, Tarbiat Modares University, Tehran, Iran; 4 Research and Development Center of Biotechnology, Tarbiat Modares University, Tehran, Iran

**Keywords:** Colorectal cancer, Cancer-associated fibroblasts, Exosomes, microRNA, Diagnosis

## Abstract

**Background:**

This study evaluated the clinical relevance of a set of five serum-derived circulating microRNAs (miRNAs) in colorectal cancer (CRC). Additionally, we investigated the role of miR-20a-5p released by exosomes derived from cancer-associated fibroblasts (CAFs) in the context of CRC.

**Methods:**

The expression levels of five circulating serum-derived miRNAs (miR-20a-5p, miR-122-5p, miR-139-3p, miR-143-5p, and miR-193a-5p) were quantified by real-time quantitative PCR (RT-qPCR), and their associations with clinicopathological characteristics in CRC patients were assessed. The diagnostic accuracy of these miRNAs was determined through Receiver Operating Characteristic (ROC) curve analysis. CAFs and normal fibroblasts (NFs) were isolated from tissue samples, and subsequently, exosomes derived from these cells were isolated and meticulously characterized using electron microscopy and Western blotting. The cellular internalization of fluorescent-labeled exosomes was visualized by confocal microscopy. Gain- and loss-of-function experiments were conducted to elucidate the oncogenic role of miR-20a-5p transferred by exosomes derived from CAFs in CRC progression. The underlying mechanisms were uncovered through luciferase reporter assay, Western blotting, enzyme-linked immunosorbent assays, as well as proliferation and migration assays.

**Results:**

The expression levels of serum-derived circulating miR-20a-5p and miR-122-5p were significantly higher in CRC and were positively correlated with advanced stages of tumorigenesis and lymph node metastasis (LNM). In contrast, circulating miR-139-3p, miR-143-5p, and miR-193a-5p were down-regulated in CRC and associated with early tumorigenesis. Except for miR-139-3p, they showed a negative correlation with LNM status. Among the candidate miRNAs, significantly elevated levels of miR-20a-5p were observed in both cellular and exosomal fractions of CAFs. Our findings indicated that miR-20a-5p induces the expression of EMT markers, partly by targeting PTEN. Exosomal miR-20a secreted by CAFs emerged as a key factor enhancing the proliferation and migration of CRC cells. The inhibition of miR-20a impaired the proliferative and migratory potential of CAF-derived exosomes in SW480 CRC cells, suggesting that the oncogenic effects of CAF-derived exosomes are mediated through the exosomal transfer of miR-20a. Furthermore**,** exosomes originating from CAFs induced increased nuclear translocation of the NF-kB p65 transcription factor in SW480 CRC cells, leading to increased interleukin-6 (IL-6) production.

**Conclusions:**

We established a set of five circulating miRNAs as a non-invasive biomarker for CRC diagnosis. Additionally, our findings shed light on the intricate mechanisms underpinning the oncogenic impacts of CAF-derived exosomes and underscore the pivotal role of miR-20a-5p in CRC progression.

**Supplementary Information:**

The online version contains supplementary material available at 10.1186/s12885-024-12190-0.

## Background

Ranked as the third most frequently detected cancer worldwide, colorectal cancer (CRC) also holds the position of the second most significant contributor to cancer-related mortalities. The prevalence has risen worldwide recently, particularly in low- and middle-income countries, underscoring the imperative need for enhanced approaches to early detection, prevention, and treatment of this disorder [1, 2]. While certain strategies have shown promise in addressing CRC, the overall prognosis for this condition remains unfavorable [3, 4]. Roughly one-third of individuals diagnosed with CRC ultimately experience the development of metastatic disease and face a notable risk of recurrence following surgical intervention [5]. This highlights the requirement for innovative biomarkers for diagnosis and prognosis that offer sufficient sensitivity and specificity.

Investigation into microRNAs (also known as miRNAs or miRs) as promising novel biomarkers for tracking tumor evolution and advancement is gaining ground [6]. miRNAs—short, endogenous, non-coding RNA molecules—function to down-regulate the expression of protein-coding genes by binding to the 3’-untraslanted region (3’-UTR) of messenger RNAs (mRNAs). miRNAs also exert control over the expression levels of a vast majority of protein-coding genes within human cells, thereby impacting nearly all developmental processes and diseases observed in humans. On the other side, the dysregulation of miRNAs contributes to the initiation and progression of cancer, as evidenced by the presence of aberrant miRNA expression in the majority, if not all, forms of malignancies [7, 8]. Interestingly, profiling the expression pattern of miRNAs will be useful to effectively categorize tumors with precision and forecast the prognosis. In fact, the remarkable stability of miRNAs in biological fluids, their straightforward extraction and quantification, along with established sensitivity and specificity, render them exceptionally well-suited for biomarker research, although further studies are needed to discover the roles of miRNAs in human cancers. Within this framework, numerous investigations have emphasized the utility of serum-derived circulating miRNAs as reliable biomarkers for the early detection of CRC [9, 10].

Recently, exosomes have gained recognition as a pivotal element in the study of cancer progression. These are distinctive extracellular vesicles, ranging in size from 30 to 100 nm, that are released from most types of cells [11, 12]. Exosomes encapsulate proteins, lipids, DNA, mRNA, and miRNAs, constituting a significant focus of interest. Notably, a substantial portion of the RNA content within exosomes consists of miRNAs, which have significant impacts on targeted cells [13]. The tumor microenvironment (TME) is a complex entity composed of different stromal cells [14]. As an active stromal component of the TME, cancer-associated fibroblasts (CAFs) release exosomes that can contribute to the formation of the pre-metastatic microenvironment and support the survival and growth of tumor cells [15]. Exosomal miRNAs released by CAFs can be transferred to recipient tumor cells and affect gene expression [16]. However, our understanding of the precise role of miRNAs secreted by CAFs in the process of tumor progression remains elusive.

In this study, our primary aim was to investigate the expression profiles of a specific set of five serum-derived circulating miRNAs (miR-122-5p, miR-139-3p, miR-143-5p, miR-193a-5p, and miR-20a-5p) in individuals diagnosed with CRC. Additionally, we sought to explore potential associations between the expression levels of these miRNAs and clinical features in CRC patients. Furthermore, our study aimed to elucidate whether exosomal miR-20a-5p secreted from CAFs promotes CRC progression by regulating the phosphatase and tensin homolog (*PTEN*) gene, thereby shedding light on the functional roles of miR-20a-5p in CRC tumorigenesis. We also investigated whether CAF-derived exosomes may result in the activation of the transcription factor NF-κB in CRC cells. Our findings proposed that the increased nuclear translocation of NF-κB p65 in CAF exosome-treated CRC cells may be associated with elevated production of interlukin-6 (IL-6).

## Materials and methods

### Functional enrichment analyses

In order to elucidate the regulatory roles of miRNAs and identify significant molecular pathways from the Kyoto Encyclopedia of Genes and Genomes (KEGG) [17], we utilized the DNA Intelligent Analysis (DIANA)-miRPath v3.0 software [18]. Additionally, for the identification of validated target pathways associated with candidate miRNAs, we employed the miRTarBase v7.0 algorithm [19]. As a standard practice, we established a threshold of *P*-value < 0.05, and the false discovery rate (FDR) was determined using Fisher's exact test. To retrieve robust experimentally supported candidate miRNAs, we utilized the miRTargetLink Human algorithm [20], which also facilitated the extraction of the interaction network.

### Subject recruitment and sample processing

The study included a cohort of forty-five patients diagnosed with CRC and having primary tumors, recruited from Shariati and Rasoul Akram Hospitals in Tehran, Iran. Additionally, a group of healthy controls, consisting of 40 individuals matched in terms of age and sex, with no prior history of malignancies or chronic diseases, were also enrolled. The study received approval from the ethics committee of Islamic Azad University, and all participants provided written informed consent. Confirmation of CRC diagnoses was established through histopathological analyses conducted on surgically resected tumor samples. Tumor stages in CRC patients were determined using the staging system based on Tumor Node Metastasis (TNM) criteria outlined by the American Joint Committee on Cancer and the Union for International Cancer Control [21]. The clinicopathological characteristics of the patients are comprehensively documented in Supplementary Table 1. To isolate serum samples, approximately 5 mL of peripheral blood was collected from each participant and subsequently subjected to centrifugation at 1,000 × g for 10 min at 4 °C. The obtained sera were then stored at -80 °C for further analysis. To isolate stromal fibroblasts, primary tissues were obtained from three CRC patients with locally advanced stage III tumors who had undergone surgery. These patients had received no prior chemo- or radio-therapy. Primary normal colon fibroblasts (NFs) were isolated from the paired adjacent non-tumor tissues.

### Isolation and culture of primary fibroblasts

Fibroblasts were enzymatically isolated from both tumor and non-tumor CRC tissues using collagenase A, following the established protocol [22]. These cells were cultured in Dulbecco’s Modified Eagle’s medium nutrient mixture F12 (DMEM/F12) supplemented with 10% fetal bovine serum (FBS). Fibroblasts within the first six passages were utilized for this study. Culture supernatants were collected when the fibroblasts reached over 70% confluency. Subsequently, the collected supernatants were centrifuged and stored at -80 °C until further use.

### Isolation and characterization of exosomes

Exosomes were isolated from the conditioned media of isolated fibroblasts by differential centrifugation as we previously described [23]. Briefly, cells were cultured in a medium containing exosome-depleted FBS at 37 °C with 5% CO2. The cell supernatants were collected and subjected to centrifugation at 300 × g for 10 min to pellet cells, followed by centrifugation at 10,000 × g for 30 min at 4 °C to further remove dead cells and debris. The resulting supernatant was then filtered through a 0.22-μm filter to exclude microvesicles larger than 200 nm. The filtered supernatant underwent an additional ultracentrifugation step at 100,000 × g for 70 min at 4 °C. To ensure the removal of any remaining protein contamination, the initial exosome pellet was resuspended in PBS and subjected to another round of ultracentrifugation. The resulting exosome-enriched pellets were reconstituted in a small volume of PBS or lysis buffer as required. The intact exosomes suspended in PBS were then stored at -80 °C for future experiments.

The morphology of purified exosomes was observed using a scanning electron microscopy (SEM). To accomplish this, a portion of the exosome suspension was fixed with 2.5% glutaraldehyde and applied onto 200-mesh Formvar-coated grids. Subsequently, the grids were stained using uranyl acetate and lead citrate before being examined using a Digital SEM, KYKYEM3200, China. The size range of the exosomes was determined using a Malvern Zetasizer Nano ZS90 (Malvern, UK), following the manufacturer's instructions. The protein concentration in exosome preparations was quantified using a BCA protein assay kit (Pierce Chemical Co., Rockford IL). The presence of the exosome-specific protein CD9 was detected in exosome preparations using a western blotting assay.

### Cellular internalization of purified exosomes

Fibroblast-derived exosomes were labeled with PKH26 (Sigma Aldrich, USA) following established protocols. Subsequently, the labeled exosomes were introduced to a subconfluent layer of CRC cells and incubated at 37 °C for 12 h. After incubation, the cells were rinsed thrice with PBS and fixed with 4% paraformaldehyde. For nuclear staining, 4′-6-diamidino-2-phenylindole (DAPI) was used. The cellular internalization of labeled exosomes was visualized using a spectral confocal microscope (Nikon Eclipse TiE, Japan).

### Transfection assays

CRC cells were transfected with either miR-20a mimic or a negative control (25 nM), as well as miR-20a-5p inhibitor or inhibitor negative control (50 nM), using Lipofectamine® 2000 (Thermo Fisher Scientific, USA). The miR-20a-5p mimic, miR-20a-5p inhibitor and their corresponding negative controls were obtained from GenePharma Co., Ltd. and the sequences were as follows: miR-20a-5p mimic, sense: 5′-UAAAGUGCUUAUAGUGCAGGUAG-3′; miR-20a-5p mimic, antisense: 5′-ACCUGCACUAUAAGCACUUUAUU-3′; miRNA negative control: 5′-UUGUACUACACAAAAGUACUG-3′; miR-20a-5p inhibitor: 5′-CUACCUGCACUAUAAGCACUUUA-3′; inhibitor negative control: 5′-UGACUGUACUGAACUCGACUG-3′. Additionally, two independent siRNAs targeting PTEN or a negative control siRNA (si-NC; obtained from GenePharma) were introduced into the cells using Lipofectamine®2000 (Thermo Fisher Scientific, USA). For the luciferase reporter assay, CRC cells were co-transfected with psi-CHECK2 luciferase reporter vector containing the 3′-UTR of PTEN and miR-20a-5p mimic or the negative control. After 48 h, the relative dual-luciferase activity was measured using the dual luciferase reporter assay system (Promega, USA).

### RNA extraction and real-time quantitative PCR

Total RNA extraction from cells, patient serum, and isolated exosomes was performed using the TRIzol reagent (Invitrogen). Subsequently, 1 μg of total RNA was reverse transcribed into cDNA using the PrimeScript 1st Strand cDNA Synthesis kit (TAKARA, Japan). For miRNA quantification, the extracted total RNA underwent polyadenylation with polyA polymerase enzyme (NEB). Then, cDNA synthesis was carried out using an anchored oligo (dT) primer, following a previously described method [24]. Reverse transcription quantitative PCR (RT-qPCR) was conducted on an ABI Step One Detection System (Applied Biosystems, USA). Relative expression levels were normalized to U6 small nuclear RNA (snRNA) and glyceraldehyde-3-phosphate dehydrogenase (GAPDH). Additionally, cel-miR-39 served as an internal reference for analyzing exosomal miRNA expression. Data analysis was performed using the 2^−ΔCt^ method, where ΔCt is calculated as Ct (Target)—Ct (Reference), and fold changes were determined using the 2^−ΔΔCt^ method [25].

### NF-κB activation assay

To evaluate NF-κB activity, nuclear and cytosolic fractions were separated utilizing a NF-κB Activation Assay Kit (FIVEphoton Biochemicals, San Diego, CA, USA) following the manufacturer’s guidelines. Subsequently, protein concentrations in the lysates were quantified using the Bradford assay, and the NF-κB p65 protein level in the nuclear and cytoplasmic preparations was detected by western blotting.

### Western blot assay

Cellular or exosomal total proteins were extracted using radioimmunoprecipitation assay (RIPA) lysis buffer (Sigma-Aldrich, USA), separated on 10–12% SDS–polyacrylamide gels (SDS-PAGE) and transmitted to a polyvinylidene difluoride (PVDF) membrane. To prevent non-specific binding, a solution of 5% bovine serum albumin (Merck) in TBST (10 mM Tris-buffered saline with 0.05% Tween 20) was employed. The blots were then probed with specific primary antibodies, appropriately diluted in TBST (1:1000). Following rinsing, the blots were treated with horseradish peroxidase (HRP)-conjugated secondary antibodies and then subjected to chemiluminescence (ECL, Amersham, Buckinghamshire, UK) for visualization. β-actin was utilized as a loading control.

### Enzyme-linked immunosorbent assay

The IL-6 levels in the supernatant of CRC cells were quantitatively measured after incubating them with 100 µg/mL exosomes derived from CAFs or NFs for 48 h, using enzyme-linked immunosorbent assay (ELISA). The cell culture supernatants were used undiluted for the assay.

### Proliferation assay

SW480 CRC cells were seeded at a density of 2 × 10^4^ cells/mL and then incubated with different concentrations of CAF-derived exosomes (25, 50, 100 µg/mL), NF-derived exosomes (100 µg/mL) or transfected with a miR-20a inhibitor. After 48 h of incubation or transfection, the viability of the cells was evaluated in triplicate using the trypan blue exclusion assay, and the results were compared to corresponding control cells.

### Transwell migration assay

SW480 CRC cells were suspended in serum-starved media and subsequently seeded into transwell chambers with 8 μm pore size (BD Biosciences). In the lower chamber, a medium containing 10% FBS served as a chemoattractant. Following a 24-h incubation period, the cells that migrated through the membrane and adhered to its lower surface were stained with 1% crystal violet (ThermoFisher Scientific) dissolved in methanol and then quantified using a light microscope.

### Statistical analysis

The data are presented as means ± standard deviation (SD) and were subjected to analysis using two-tailed Student's t-tests or one-way ANOVAs, as deemed appropriate for the respective comparisons. A significance threshold of *p*-value < 0.05 was utilized for this study. The diagnostic accuracy of the candidate serum-derived miRNAs was evaluated using a receiver operating characteristic (ROC) curve test, which involved calculating the area under the curve (AUC) along with 95% confidence intervals. All statistical analyses as well as visualization were carried out using GraphPad Prism version 9.0 (GraphPad Software, Inc., La Jolla, CA, USA).

## Results

### Expression levels of CRC-secreted miRNAs and their possible correlation with clinical characteristics

**Fig. 1:**
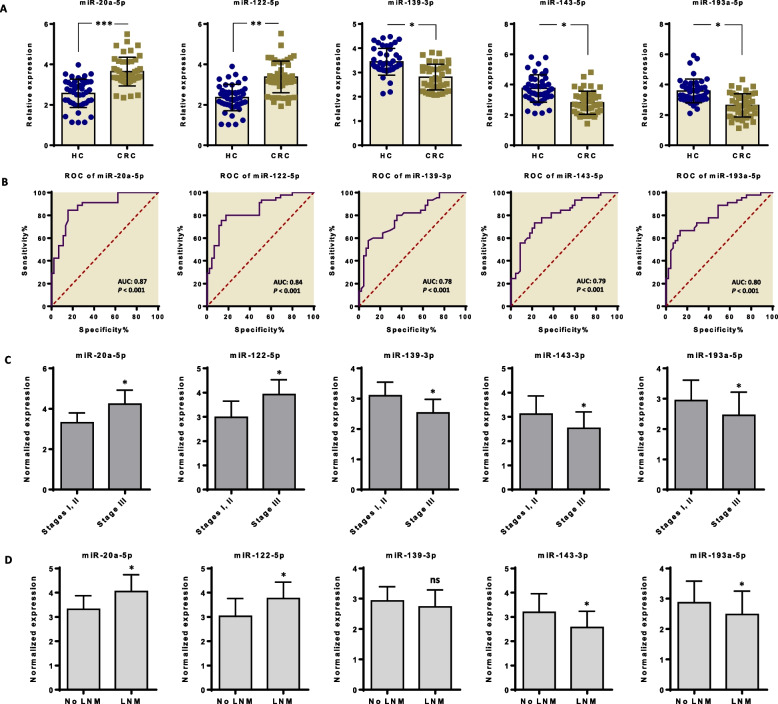
A panel of five serum-derived circulating miRNAs and their potential for diagnosing CRC.** A** Expression levels of serum-derived circulating miRNAs in CRC patients compared to healthy controls. Circulating miR-20a-5p and miR-122-5p were significantly higher in CRC serum samples compared to healthy controls. In contrast, miR-139-3p, miR-143-5p, and miR-193a-5p were lower in CRC serum samples than in normal samples. **B** Receiver operating characteristic (ROC) curve analysis was used to assess whether the candidate miRNAs can be potential biomarkers for CRC diagnosis. All miRNAs exhibited satisfactory Area Under the Curve (AUC) values, with miR-20a-5p at 0.87, miR-122-5p at 0.84, miR-139-3p at 0.78, miR-143-5p at 0.79, and miR-193a-5p at 0.80. Crucially, these AUC values held significant statistical significance (all *p*-values are < 0.001), affirming the capacity of these miRNAs to successfully distinguish between CRC and healthy groups. **C** The expression levels of circulating miRNAs in association with TNM stage I-III. While the expression levels of miR-20a-5p and miR-122-5p were higher in advanced stages of tumorigenesis, other miRNAs (i.e., miR-139.30, miR-143-3p, and miR-193a-5p) showed a positive correlation with early stages of tumorigenesis. **D** The expression levels of circulating miRNAs in CRC patients with positive and negative lymph node metastasis (LNM). While high expression of miR-20a-5p and miR-122-5p was found in LNM positive patients, the expression levels of miR-143-3p and miR-193a-5p were higher in patients without LNM. No significant differences were found for miR-139-3p between positive and negative LNM status

To assess the diagnostic potential of the miRNA signature, we analyzed the expression of each candidate miRNA in serum samples obtained from individuals with CRC and those without any gastrointestinal symptoms (healthy individuals). RT-qPCR was employed to profile the miRNA expression patterns. Our findings indicated that the expression levels of miR-122-5p and miR-20a-5p were elevated in serum samples derived from CRC patients compared to the healthy controls. Conversely, the expression levels of circulating miR-139-3p, miR-143-5p, and miR-193a-5p were decreased in CRC patients than normal samples (Fig. 1A). We subsequently investigated the utility of these miRNAs as potential biomarkers for CRC detection using ROC curve analysis. The results demonstrated that all miRNAs exhibited satisfactory AUC values, with miR-20a-5p at 0.87, miR-122-5p at 0.84, miR-139-3p at 0.78, miR-143-5p at 0.79, and miR-193a-5p at 0.80. Importantly, these AUC values were statistically significant (all *p*-values < 0.001), indicating that these miRNAs can effectively differentiate between the CRC and healthy groups (Fig. 1B). We further investigated whether the expression of candidate miRNAs was associated with the TNM score. Intriguingly, miR-20a-5p and miR-122-5p exhibited correlations with advanced stages of tumorigenesis. Conversely, the expression levels of miR-139-3p, miR-143-5p, and miR-193a-5p were found to decrease in advanced stages of CRC, while positively correlating with the earlier stages of tumorigenesis (TNM stages I and II) (Fig. 1C). Furthermore, in order to elucidate the clinical relevance, we initially compared the expression levels of these miRNAs with LNM status in CRC patients. Our findings revealed that miR-20a-5p and miR-122-5p exhibited increased expression in patients with LNM, whereas miR-143-5p and miR-193a-5p demonstrated decreased expression in LNM-positive patients. Interestingly, with regards to miR-139-3p, we did not observe a significant change in its levels between CRC patients and healthy individuals, suggesting that this miRNA is independent of LNM status (Fig. 1D).

### Functional enrichment analysis

By utilizing the 'genes union analysis' function within DIANA-miRPath v.3.0, we uncovered potential roles for miR-20a-5p and miR-122-5p not only in CRC but also in renal cell carcinoma and pancreatic cancer. Importantly, miR-20a-5p showed a high rank of association with pathophysiological features (Supplementary Fig. 1A). To ensure robustness, we employed CancerMIRNome (http://bioinfo.jialab-ucr.org/CancerMIRNome/) to assess gene expression patterns across various cancer types, including CRC. The results showed that there was a consistent increase in the expression patterns of miR-20a and miR-122 in CRC patients. However, it is important to note that while miR-122 displayed a higher trend in patients, this increase was not statistically significant. Conversely, miR-139 and miR-193 displayed decreased expression patterns in these patients. However, there was a contradictory finding regarding miR-143, which demonstrated an increase in expression among CRC patients, although our data did not support this finding (Supplementary Fig. 1B).

### Characterization and cellular uptake of exosomes derived from primary fibroblasts

**Fig. 2:**
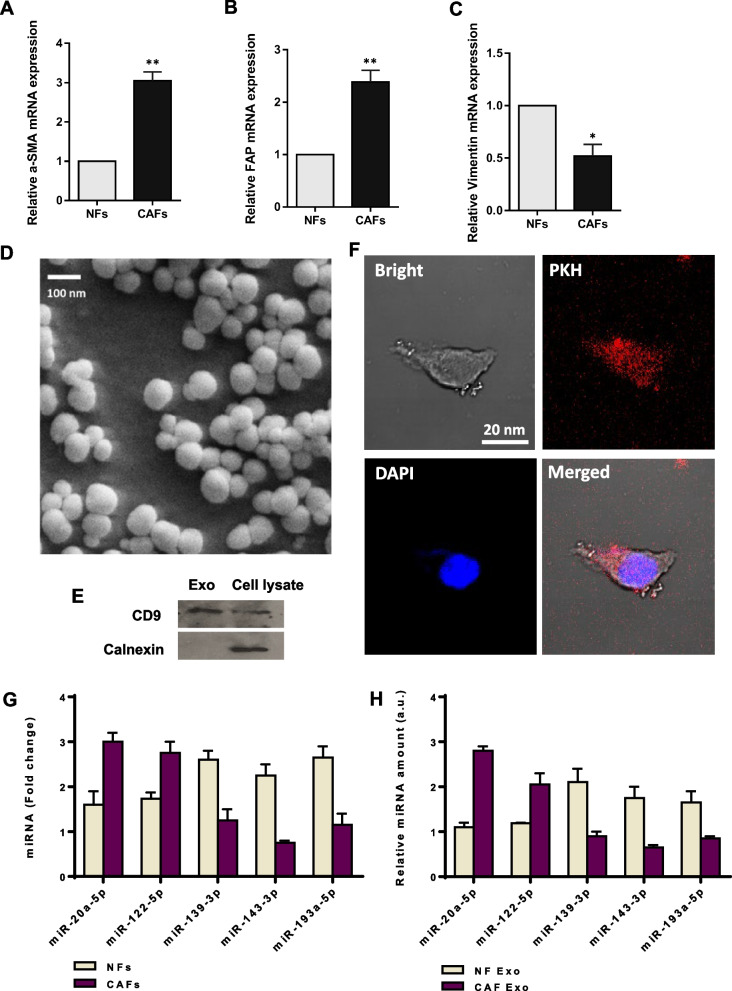
Exosomes derived from isolated CAFs from CRC tissues contains miR-20a. **A-C** The identity of the CAFs was confirmed through the detection of the CAF surface markers Alpha Smooth Muscle Actin (α-SMA) and Fibroblast Activation Protein (FAP), as well as the fibroblast marker Vimentin, using RT-qPCR. The positive expression of α-SMA and FAP in the isolated and cultured cells strongly indicated the successful isolation of CAFs (*p* < 0.01), while higher levels of Vimentin were observed in NFs (*p* < 0.05). **D** The purified exosomes derived from CAFs and NFs displayed a spherical morphology, with their diameters falling within the range of 50 to 150 nm. This was verified through scanning electron microscopy (SEM) imaging. **E** Western blot analysis showed high expression of CD9 as an exosome-specific surface marker. The cytoplasmic protein marker Calnexin was present in the entire cell lysate but was absent in the isolated exosomes, suggesting that the exosome samples were free from contamination with other vesicles, such as those from the endoplasmic reticulum. The cropped blots are presented correspondingly. Full-length blots are presented in Supplementary Fig. 3. **F** Analysis using confocal microscopy unveiled the presence of red fluorescence of PKH26-labeled exosomes within the cytoplasm and perinuclear region of the recipient SW480 CRC cells, suggesting successful internalization of exosomes in CRC cells. The nuclear staining was carried out by DAPI. The bar represents 20 μm. **G**, **H** The expression levels of five candidate miRNAs in CAFs, NFs, and their corresponding exosomes. According to RT-qPCR results, miR-20a was found to be higher in CAFs (**G**) and their exosomes (**H**), when compared to NFs and their respective exosomes. All experiments were conducted in triplicate. NF Exo: NF-derived exosomes, CAF Exo: CAF-derived exosomes. Columns represent means of three different experiments; bars represent SD. *P < 0.05, **P < 0.01

The tissue samples from each patient were meticulously dissected, and regions containing CAF and NF were carefully sectioned into small, separate pieces. These cells were then sorted, cultured, and distinguished based on their morphology and biomarker expression. Notably, the cultured cells displayed the typical cellular morphology of CAFs, characterized by a long spindle shape, multilayered growth, and a disordered arrangement at a specific cell density. To confirm the identity of the CAFs, we explored the CAF surface markers Alpha Smooth Muscle Actin (α-SMA) and Fibroblast Activation Protein (FAP), as well as the fibroblast marker Vimentin using RT-qPCR. The isolated and cultured cells showed significantly higher expression levels of α-SMA and FAP, indicating the successful isolation of CAFs, while NFs exhibited elevated levels of Vimentin (Fig. 2A-C).

The purified exosomes obtained from CAFs exhibited a spherical morphology with diameters falling within the range of 50 to 150 nm, as confirmed through scanning electron microscopy (SEM) imaging (Fig. 2D). Furthermore, the western blotting assay consistently demonstrated the presence of well-established exosome-specific marker CD9 in the isolated exosomes (Fig. 2E). To ensure the purity of the exosome preparation and exclude contamination with other extracellular vesicles or cellular compartments, we measured the protein levels of Calnexin, an endoplasmic reticulum marker. The results showed that Calnexin was not detected in the isolated exosomes, further confirming the purity of the exosome preparation (Fig. 2E). In addition, to investigate the uptake of CAF-Exo by CRC cells, we prepared PKH26-labeled exosomes isolated from CAFs. These labeled exosomes were then incubated with SW480 CRC cells at a concentration of 100 µg/mL for 12 h. Confocal microscopy analysis revealed the presence of red fluorescence within the cytoplasm and perinuclear region of the recipient cells, providing clear evidence of the successful internalization of CAF-derived exosomes in CRC cells (Fig. 2F).

### The expression levels of candidate miRNAs in CAFs and NFs, and their corresponding exosomes

The expression levels of the candidate miRNAs were evaluated in both CAFs and NFs, as well as in their derived exosomes. The results indicated that miR-20a-5p and miR-122-5p exhibited higher expression levels in CAFs compared to NFs. Conversely, the levels of miR-139, miR-143, and miR-193 were elevated in NFs. The same findings were observed when investigating the expression levels of these miRNAs in exosomes derived from both CAFs and NFs. Essentially, the data demonstrated that miR-20a-5p and miR-122-5p were up-regulated in CAFs and their corresponding exosomes (Fig. 2G, H).

### miR-20a-5p promotes CRC cell aggressiveness via EMT initiation partly through targeting PTEN

**Fig. 3:**
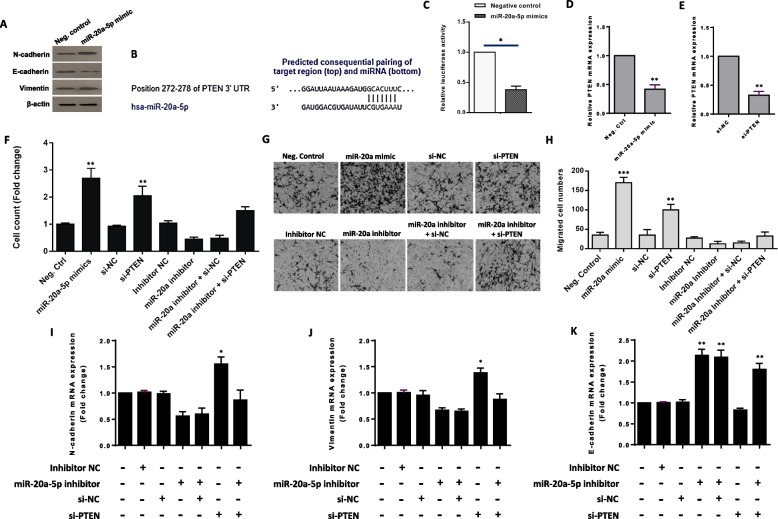
miR-20a-5p enhances CRC cell proliferation and migration, and up-regulates Epithelial-Mesenchymal Transition (EMT) markers partly through targeting PTEN. **A** Western blot analysis indicated that while the expression levels of N-cadherin and Vimentin significantly increased after miR-20a-5p mimic transfection, there was a notable decrease in the protein level of E-cadherin following the transfection. β-actin was employed as an endogenous loading control. The cropped blots are presented correspondingly. Full-length blots are presented in Supplementary Fig. 4. **B** TargetScan 7.2 predicted the complementary binding site between miR-20a-5p and PTEN's 3'-UTR (http://www.targetscan.org). **C** A luciferase assay demonstrated a significant reduction in luciferase activity upon introducing the miR-20a-5p mimic, indicating the binding affinity between miR-20a-5p and PTEN's 3'-UTR. **D**,** E** The transcript expression level of PTEN was assessed following the use of both the miR-20a-5p mimic and siRNA targeting PTEN. In both cases, PTEN expression decreased at the mRNA level, and a similar pattern was achieved. **F** Transfection of miR-20a-5p mimic to SW480 CRC cells led to an observable increase in the cell count, compared to the negative control group. Notably, transfection of CRC cells with si-PTEN had a similar effect and enhanced CRC cell proliferation. Importantly, the suppression of miR-20a-5p with its specific inhibitor effectively decreased cell numbers. However, the inhibitory effect of the miR-20a-5p inhibitor on SW480 CRC cell proliferation was partially rescued when SW480 CRC cells were concurrently transfected with si-PTEN. **G** Representative photomicrographs of the migration potential of SW480 CRC cells in different conditions after 48 h of transfection, assessed using the transwell assay. **H** Quantitative assessment of migrated cells revealed that the transfection of CRC cells with either the miR-20a-5p mimic or si-PTEN can give rise to substantial increase in the migration rate of CRC cells. Conversely, the miR-20a-5p inhibitor resulted in a reduction in the number of migrated cells. Of significant importance**,** the suppressive effect of the miR-20a-5p inhibitor on SW480 CRC cell migration was partially diminished when SW480 CRC cells were concurrently transfected with si-PTEN. **I**,** J** The mean normalized ratios measured for EMT-related markers N-cadherin and Vimentin indicated that transfection of miR-20a-5p inhibitor led to reduced expression levels of each marker. Importantly, introduction of si-PTEN into SW480 CRC cells caused a significant up-regulation in the transcript expression levels of N-cadherin and Vimentin. **K** The mean normalized ratios measured for E-cadherin showed that transfection of the miR-20a-5p inhibitor led to increased expression levels of E-cadherin. Transfection of si-PTEN into SW480 CRC cells caused a down-regulation in the transcript expression level of E-cadherin to some extent. However, when si-PTEN was introduced concurrently with the miR-20a-5p inhibitor, it simultaneously resulted in promoting the expression of E-Cadherin. Columns represent means of three different experiments; bars represent SD. *P < 0.05, **P < 0.01

Epithelial-to-mesenchymal transition (EMT), a fundamental phenotypic transformation observed in embryonic development, tissue remodeling, and wound healing, holds a pivotal role in facilitating tumor invasion and metastasis, as noted in studies [26]. This process is dynamic and reversible and frequently takes place at the invasive edge of various metastatic cancers, including CRC [27]. To determine any connection between the miR-20a-5p-induced promotion of EMT in CRC cells, the expression levels of EMT-related markers including N-cadherin, E-cadherin, and Vimentin were measured using Western blot assays. The results showed that the protein levels of N-cadherin and Vimentin were significantly increased after miR-20a-5p overexpression (by introducing the miR-20a-5p mimic). On the other hand, the protein level of E-cadherin decreased following miR-20a mimic transfection (Fig. 3A). In essence, these results suggest that miR-20a-5p may regulate the expression of genes associated with EMT.

Subsequently, we used the public database TargetScan to search for potential target genes of miR-20a-5p. *PTEN* was identified as one of the top candidates that can per se inhibit metastasis and EMT [28, 29] (Fig. 3B). To examine the binding affinity between miR-20-5p and the 3'-UTR of *PTEN*, a luciferase assay was conducted. The outcomes unequivocally revealed that the introduction of the miR-20a-5p mimic resulted in a substantial reduction in luciferase activity, suggesting that PTEN is indeed a direct target gene of miR-20a-5p (Fig. 3C). To be on the safe side and in order to test the regulatory effect of miR-20a-5p on *PTEN*, we checked the gene expression of *PTEN* after using miR-20a-5p mimic; the results supportably showed that the expression of *PTEN* decreased in mRNA levels (Fig. 3D).

Next, we conducted a series of in vitro experiments to investigate whether PTEN plays a role in the process of EMT induced by miR-20a-5p in CRC cells. To eliminate the possibility that PTEN was the sole gene affected by miR-20a, as other genes might also be impacted by this miRNA, we employed a specific siRNA designed exclusively to target PTEN. As shown in Fig. 3E, PTEN transcript level was decreased after transfection with a siRNA against PTEN (si-PTEN). We also found that the transfection of miR-20a-5p mimic to SW480 CRC cells led to an observable increase in the cell count; however, no changes were observed when the cells were transfected with negative control (Fig. 3F). As shown earlier, the inhibition of PTEN by miR-20a-5p occurred, and we found that the implementation of siRNA specifically targeting PTEN yielded similar results (Fig. 3F). Importantly, the inhibition of miR-20a-5p with its specific inhibitor effectively led to a decrease in cell numbers. Notably, we observed that the concurrent addition of the miR-20a-5p inhibitor and si-NC decreased the cell counts; however, of significant importance, the co-transfection of SW480 cells with both the miR-20a-5p inhibitor and si-PTEN resulted in a relatively increase in the cell numbers (Fig. 3F). In essence, this observation underscores the intricate interplay between miR-20a-5p and PTEN in regulating cell proliferation. Furthermore, the transwell invasion assay revealed that the transfection of SW480 CRC cells with either the miR-20a-5p mimic or si-PTEN led to a substantial increase in the migration rate of CRC cells. Conversely, the miR-20a-5p inhibitor resulted in a reduction in the number of migrated cells (Fig. 3G, H). Simultaneous treatment with an inhibitor of miR-20a-5p and si-PTEN notably reinstated the migration capability of SW480 cells that had been suppressed by the miR-20a-5p inhibitor (Fig. 3G, H). These results provided compelling evidence that PTEN inhibition, partly through miR-20a-5p, is closely associated with enhanced cell migration.

As discussed earlier, E-Cadherin, N-Cadherin, and Vimentin serve as pivotal markers of EMT. To elucidate the roles of miR-20a-5p and its target gene, *PTEN*, in this context, we assessed the expression levels of the EMT markers in SW480 CRC cells. The results demonstrated that transfection of the miR-20a-5p inhibitor, either alone or in combination with si-NC, led to decreased expression levels of EMT markers (i.e., N-Cadherin and Vimentin). However, with regard to the epithelial marker E-Cadherin, its expression levels specifically increased in SW480 cells. Remarkably, we observed that the exclusive introduction of si-PTEN caused an augmentation in the expression levels N-Cadherin and Vimentin. However, when si-PTEN was introduced concurrently with the miR-20a-5p inhibitor, the inhibitory effect of the miR-20a inhibitor was diminished in SW480 CRC cells (Fig. 3I-K). In summary, the concurrent treatment with an inhibitor of miR-20a-5p and si-PTEN significantly restored the migration capability of CRC cells that had been suppressed by the miR-20a-5p inhibitor. These results provide robust evidence for the involvement of miR-20a-5p and PTEN in the regulation of EMT and cell migration in CRC.

### Exosomal transfer of miR-20a-5p from CAFs enhances colorectal cancer cell proliferation and migration

**Fig. 4:**
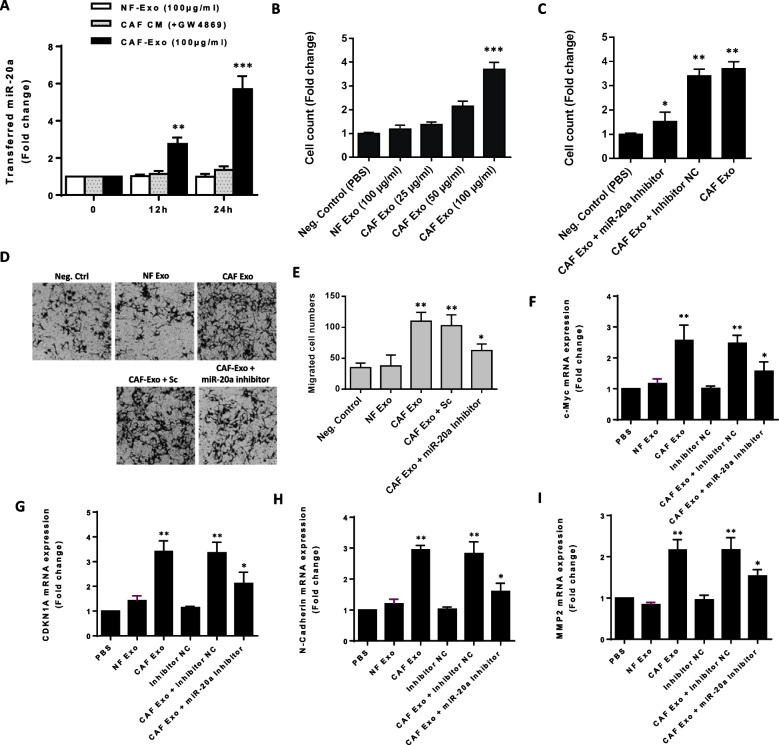
Exosomal transfer of miR-20a-5p from CAFs causes enhanced CRC cell proliferation and migration.** A** The mean normalized ratio for miR-20a-5p levels was assessed by RT-qPCR in SW480 CRC cells at 12 and 24-h time points. SW480 CRC cells were pre-treated with RNA polymerase inhibitor α-amanitin for 8 h before incubation with 100 μg/ml CAF-Exo. Inhibiting the endogenous source of miR-20a-5p using α-amanitin did not result in a significant change in the fold change of miR-20a-5p, suggesting the exosomal origin for this miRNA. The cells incubated with PBS and α-amanitin were used as a control. To confirm that this effect was solely attributed to exosomes and not influenced by any extraneous factors, we inhibited exosome secretion by introducing GW4869 in CAFs. RT-qPCR results revealed that CAF-secreted exosomal miR-20a is transferred to SW480 CRC cells in a time-dependent manner. **B** Cell proliferation exhibited a dose-dependent response, increasing as SW480 CRC cells were treated with increasing concentrations of CAF-derived exosomes. The highest level of cell proliferation was observed when the cells were treated with 100 µg/ml of CAF-derived exosomes. **C** Incubation of SW480 CRC cells with 100 μg/ml CAF-derived exosomes led to a considerable increase in the cell count, when compared to the negative control group. Importantly, transfection of miR-20a-5p inhibitor effectively diminished the promoting effect of CAF-derived exosomes on SW480 CRC cells. **D** Representative photomicrographs of the migration potential of SW480 CRC cells in different conditions after 48 h of incubation, assessed using the transwell assay.** E** Quantitative assessment of migrated cells revealed that the incubation of CRC cells with 100 μg/ml CAF-derived exosomes caused a significant increase in the migration rate of CRC cells. Of significant importance**,** the promoting effect of CAF-derived exosomes on SW480 CRC cell migration was partially diminished when SW480 CRC cells were concurrently transfected with the miR-20a-5p inhibitor. **F-I** The mean normalized ratios measured for proliferation markers (i.e., c-Myc and CDKN1A) and migration markers (N-cadherin and MMP2) indicated that incubation of SW480 CRC cells with 100 μg/ml CAF-derived exosomes led to an up-regulation in the expression levels of these markers. However, no significant changes were detected when SW480 cell were incubated with NF-derived exosomes. Notably, transfection of miR-20a-5p inhibitor into CAF Exo-treated SW480 CRC cells reduced the promoting effects of CAF-derived exosomes on the expression levels of these proliferation and migration markers. Columns represent means of three different experiments; bars represent SD. *P < 0.05, **P < 0.01, ***P < 0.001

Considering a relatively higher amount of miR-20a in exosomes derived from CAFs (Fig. 2H), we sought to investigate whether exosomes containing miR-20a released from CAFs can affect the malignant phenotype of SW480 CRC cells. To demonstrate that exosomal miR-20a is transferable into SW480 CRC cells, the cells were treated with exosomes derived from both CAFs and NFs. Intriguingly, the results demonstrated that treating SW480 cells with NF-derived exosomes at a concentration of 100 µg/ml did not show any discernible trend in the levels of miR-20a-5p. However, when SW480 cells were treated with the same quantity of CAF-derived exosomes, a time-dependent increase in miR-20a-5p levels was observed (Fig. 4A). As exosome generation and secretion can be stimulated by ceramide and repressed by the sphingomyelinase inhibitor GW4869 [30], we applied the conditioned media (CM) derived from GW4869-treated CAFs as a treatment on SW480 cells. The results unequivocally indicated no changes in the levels of miR-20a-5p within the specified time frame (Fig. 4A). In all groups, we specifically targeted transcription by introducing α-amanitin (50 µg/ml), which is an inhibitor of transcriptional activation [31] (see Supplementary Fig. 2). These findings strongly support the notion that miR-20a-5p is transferable from CAFs to CRC cells via exosomes.

To verify whether exosomal miR-20a-5p promotes CRC cell proliferation, SW480 CRC cells were treated with varying concentrations of CAF-derived exosomes (25, 50, and 100 µg/ml) for 24 h. The results demonstrated a direct correlation, where an increase in the concentration of CAF-derived exosomes led to a proportional augmentation in CRC cell proliferation (Fig. 4B). In order to assess the potential roles of NF-derived exosomes, SW480 cells were incubated with 100 µg/ml of NFs-derived exosomes. However, this experiment did not reveal any significant changes in CRC cell proliferation (Fig. 4B). These findings indicated that exosomes derived from CAFs, but not NFs, have the capacity to enhance CRC cell proliferation. To ascertain that the observed increase in cell proliferation is indeed attributed to exosomal miR-20a and not influenced by other factors, we conducted incubations involving CAF-derived exosomes along with miR-20a inhibitor. The results demonstrated that when SW480 cells were incubated with 100 µg/ml CAF-derived exosomes in combination with the miR-20a inhibitor, there was a notable decrease in cell proliferation compared to the condition where cells were treated solely with CAF exosomes (Fig. 4C). These results strongly suggest that exosomal miR-20a secreted by CAFs might be an important factor capable of enhancing CRC cell proliferation.

To establish that the observed increase in CRC cell migration is indeed attributable to exosomal miR-20a secreted by CAFs, we conducted a transwell assay involving the treatment of CRC cells with CAF-Exo, CAF-Exo along with miR-20a inhibitor, and NF-Exo. Additionally, to exclude the roles of any other candidate exosomal miRNAs, we treated SW480 cells with CAF-derived exosomes along with the miR-20a-inhibitor. The findings demonstrated that when SW480 cells were incubated with CAF-Exo, whether with or without the miR-20a inhibitor, cell migration increased. However, transfection of the miR-20a inhibitor decreased cell migration compared to the condition where cells were incubated solely with CAF-Exo (Fig. 4E). These findings emphasized the significant roles played by miR-20a shuttled by CAF-derived exosomes in enhancing CRC cell migration. Furthermore, a RT-qPCR assay was used to verify whether exosomal miR-20a-5p could affect transcript levels of proliferation and migration markers. The data indicated that up-regulation of proliferation markers (i.e., c-Myc and CDKN1A) and migration markers (N-cadherin and MMP2) were observed in SW480 CRC cells after incubation with CAF-derived exosomes. In contrast, NF-derived exosomes had no considerable effect on the transcript levels of these markers. Importantly, when SW480 cells were transfected with the miR-20a inhibitor, the enhancing effect of CAF-derived exosomes on CRC cells were to some extent diminished (Fig. 4F-I). These data suggest that exosomes-derived from CAFs have the potential to enhance CRC cell proliferation and migration, possibly through the involvement of miR-20a-5p.

### Increased nuclear translocation of NF-κB p65 in CAF Exo-treated CRC cells is associated with elevated production of IL-6

**Fig. 5:**
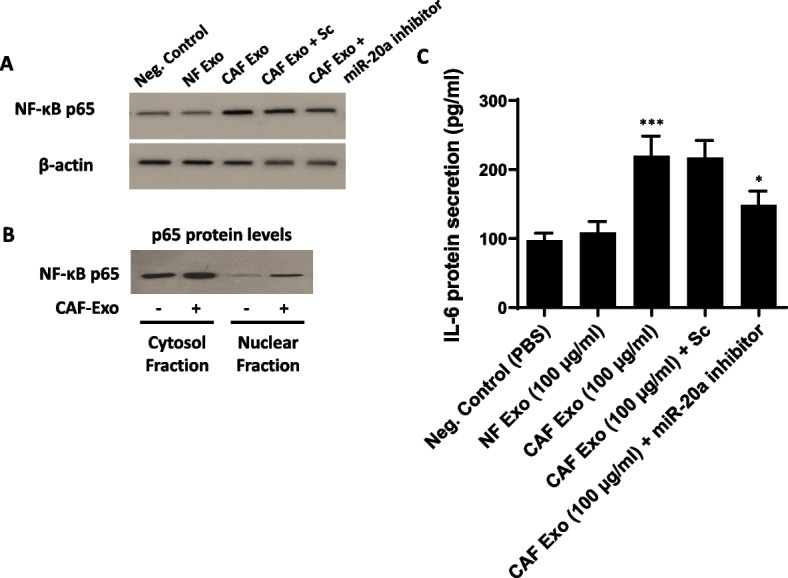
Exosomes originating from CAFs activate NF-κB p65 in CRC cells, leading to the secretion of interleukin-6.** A** Western blot analysis of NF-κB p65 protein levels in the total cell lysate of SW480 CRC cells, 48 h after incubation with 100 μg/ml exosomes derived from CAFs or NFs. Importantly, transfection of miR-20a-5p inhibitor reduced the promoting effect of CAF-derived exosomes on NF-κB p65 protein levels in SW480 CRC cells. β-actin was used as an endogenous loading control. **B** Elevated nuclear (Nuc) p65 protein levels indicated NF-κB activation after treatment with 100 µg/ml exosomes derived from CAFs. Cyt: cytoplasmic fraction. The cropped blots are presented correspondingly. Full-length blots are presented in Supplementary Fig. 5. **C** The level of interleukin-6 (IL-6) protein secretion was evaluated using ELISA. The results revealed a significant increase in IL-6 production in CRC cells stimulated with 100 µg/ml of exosomes derived from CAFs for 48 h, in comparison to cells treated with PBS or NF-derived exosomes. The inhibition of miR-20a significantly diminished the enhancement of IL-6 production in SW480 CRC cells caused by exosomes derived from CAFs. Columns represent means of three different experiments; bars represent SD. *P < 0.05, ***P < 0.001

Given the well-established role of constitutive activation of nuclear factor-κB (NF-κB) signaling in the development and progression of CRC [32], we aimed to investigate whether the transcription factor NF-κB is implicated in the paracrine effects of CAFs on CRC cells. In pursuit of this, SW480 CRC cells were subjected to incubation with 100 μg/ml of CAF-derived exosomes, and the expression of endogenous NF-κB signaling subunit p65 in the whole-cell lysate was measured and compared to the negative control cells. The results revealed a significant increase in the protein level of NF-κB p65 after 48 h of incubation with CAF-derived exosomes in SW480 cells compared to cells treated with PBS or NF-derived exosomes (Fig. 5A). Notably, the inhibition of miR-20a-5p reduced the enhancement of NF-κB p65 protein levels in SW480 CRC cells caused by CAF-derived exosomes. Considering the elevated protein levels of p65 in the total cell lysate of CRC Exo-incubated SW480 cells, we also assessed the localization of p65 protein via western blotting. Interestingly, results provide evidence of p65 translocation into the nucleus of SW480 cells following incubation with 100 μg/ml CAF Exo after 48 h (Fig. 5B). These findings strongly suggest that the NF-κB p65 transcription factor is indeed more active within the nucleus of CRC cells incubated with CAF-derived exosomes. Given that interleukin-6 (IL-6) production is associated with NF-κB activation, we investigated IL-6 protein secretion from SW480 CRC cells following treatment with 100 µg/ml CAFs- or NFs-derived exosomes. The results clearly indicated that IL-6 secretion from CRC cells stimulated with CAF-derived exosomes (100 µg/ml) for 48 h was significantly higher compared to cells treated with PBS or NF-derived exosomes (Fig. 5C). Importantly, transfection of miR-20a-5p inhibitor diminished the promoting effect of CAF-derived exosomes on IL-6 secretion from CRC cells to some extent, providing evidence to support the functional role of miR-20a-5p shuttled by CAF-derived exosomes (Fig. 5C). Overall, these findings propose a potential association between NF-κB p65 activation upon CAF-derived exosome incubation and the production of IL-6 in CRC cells.

## Discussion

CRC stands out as one of the most perilous malignancies, with its worldwide occurrence and fatality rates witnessing a substantial rise in recent decades. Despite extensive documentation of genetic and epigenetic alterations in CRC, the intricate molecular mechanisms underpinning the development of this disease still require further investigation. Additionally, the discovery of novel therapeutic targets is of utmost importance for enhancing the treatment of CRC. There is also a pressing need for non-invasive diagnostic tools to reduce the mortality rates associated with CRC [33, 34].

In recent times, the field of exosome biology and its clinical implications in oncology have garnered growing interest. Among the various types of cargo transported by exosomes, miRNAs have come under the spotlight [31, 35, 36]. Here we aimed to identify robust biomarkers for CRC and subsequently elucidate the mechanisms by which the most significant biomarker influences tumorigenesis. Our findings established a signature of five-circulating miRNAs (miR-20a-5p, miR-122-5p, miR-139-3p, miR-143-5p, and miR-193a-5p) as a novel non-invasive diagnostic biomarker for patients with CRC (Fig. 1). Consistent with our results, Selven et al. also demonstrated that high expression levels of miR-20a-5p are positively correlated with a favorable prognosis in advanced stages of CRC [37]. Additionally, miR-122-5p has been identified to promote CRC progression [38]. Therefore, these studies provide evidence of the oncogenic nature of these miRNAs in CRC. Our findings indicated that miR-20a-5p and miR-122-5p displayed associations with advanced stages of tumorigenesis, while miR-139-3p, miR-143-5p, and miR-193a-5p showed positive correlations with the earlier stages of tumorigenesis, specifically TNM stages I and II (Fig. 1). These findings collectively highlight the potential of these miRNAs as valuable biomarkers for CRC diagnosis and prognosis. However, there remains an unanswered question regarding the specific roles that these candidate miRNAs play during CRC tumorigenesis.

CAF, as a prominent component of the TME, plays a crucial role in promoting tumor progression by releasing an array of soluble factors or structurally remodeling the extracellular matrix [39]. Additionally, these stromal cells can induce a more malignant activity of tumor cells through the release of exosomes that eventually promote tumor progression [40]. The impact of exosomes on CRC progression has been extensively documented. Notably, CAF-derived exosomes can enhance metastasis and confer resistance to chemotherapy in CRC [41, 42]. CAFs and NFs exhibit distinct variations in terms of their morphology and gene expression [42]. CAFs stand out as the predominant stromal components and hold crucial roles in shaping the TME and impacting the behavior of tumor cells, primarily through the release of proteolytic enzymes, growth factors, and cytokines [43]. While CAFs have historically been categorized as α-SMA + myofibroblasts [44], various other markers, e.g., FAP, fibroblast-specific protein 1 (FSP1), and platelet-derived growth factor receptor α (PDGFRα) have been documented as well [45]. Our data consistently revealed an up-regulation in the expression levels of *α-SMA* and *FAP* within the isolated CAFs. Conversely, NFs exhibited elevated expression of Vimentin, a marker highly expressed in fibroblasts (Fig. 2A). At the next step, we showed that the expression analysis of candidate miRNAs in both CAFs and NFs exhibited a comparable pattern in patients’ samples. Specifically, miR-20a-5p and miR-122-5p were found to be elevated in CAFs, while miR-139-3p, miR-143-5p, and miR-193a-5p displayed increased expression levels in NFs (Fig. 2G). Subsequent analysis revealed a consistent expression pattern in exosomes derived from both CAFs and NFs. However, concerning miR-20a-5p, we identified notably higher expression levels in exosomes derived from CAFs compared to the other candidates (Fig. 2H). Out of all the candidate miRNAs, we concentrated our attention on miR-20a-5p for several key reasons: firstly, it displayed characteristics consistent with oncomiRs; secondly, it exhibited the most robust correlation with cancer-related features identified through functional enrichment analyses; and thirdly, its expression level was significantly higher than that of any other candidate miRNAs in both CAFs and the exosomes derived from CAFs.

As cancer progresses, tumor cells undergo processes such as detachment from the basement membrane, invasion into nearby tissues, and the establishment of colonies in distant locations. EMT plays a crucial role in regulating these processes, particularly in metastasis [46]. In the context of CRC, miR-20a-5p has been identified to induce EMT, indicating its oncogenic role. Interestingly, miR-20a-5p's function seems to vary based on tissue context; for example, in endometrial cancer, miR-20a-5p inhibits EMT by targeting STAT3 [47]. However, in CRC, miR-20a-5p promotes EMT, suggesting its tissue-specific function. Here, we showed a correlation between elevated levels of miR-20a-5p and increased aggressiveness of CRC cells. PTEN, a well-known tumor suppressor gene frequently disrupted in cancers, including CRC, is often silenced at the transcriptional level [48]. Various studies have highlighted the regulation of PTEN by miRNAs in different cancer types [49]. Given that miR-20a directly targets PTEN (Fig. 3B, C), we speculated whether the exosomal transfer of miR-20a secreted from CAFs might facilitate CRC cell progression partly through targeting PTEN. We identified a reduction in *PTEN* expression in cells transfected with miR-20a-5p mimic (Fig. 3D). Consistently, Fang et al. [50] identified that miR-20a-5p contributes to hepatic glycogen synthesis through targeting p63 to modulate p53 and *PTEN* expression. Notably, the miR-20a-5p/PTEN axis was also identified by Zhang et al., who proposed that the up-regulation of miR-20a-5p enhances hepatocyte proliferation by targeting the PTEN/AKT signaling pathway [51], aligning with our findings. Our study revealed a close association between PTEN inhibition and increased CRC cell proliferation and migration, a phenomenon partially attributable to the actions of miR-20a-5p (Fig. 3F-H). Notably, Coronel-Hernández et al. [52] made a similar observation regarding miR-26a's ability to reduce PTEN levels in CRC cells, a process that correlates positively with heightened rates of cell proliferation and migration. Furthermore, our study revealed that inhibiting PTEN led to increased expression levels of EMT markers in CRC cells (Fig. 3I-K). Consistently, our data revealed that miR-20a-5p can induce EMT markers in CRC cells, partly through PTEN inhibition, and enhance the expression of E-Cadherin—an epithelial marker for EMT. These findings are particularly intriguing and add depth to our understanding of the role of miR-20a-5p in CRC, suggesting a potential mechanism for its oncogenic function, ultimately driving elevated cell proliferation and migration in CRC.

Exosomal transfer of miRNAs is instrumental in modulating the TME. Our research focused on understanding the intercellular communication mediated by exosomes within the context of the CRC microenvironment. Our data demonstrated that miR-20a was enriched in CAF-derived exosomes and was capable of being transferred as cargo to CRC cells (Fig. 4A). Importantly, the exosomal transfer of miR-20a-5p secreted from CAFs was associated with enhanced proliferation and migration rates of CRC cells (Fig. 4C-E). Thus, it can be inferred that exosomal miR-20a released by CAFs potentially enhances the aggressive behavior of CRC cells. Our findings further indicated that exosomes originating from CAFs led to heightened levels of the NF-kB p65 transcription factor. Consequently, NF-κB p65 exhibited increased activity within the nuclei of CRC cells treated with exosomes derived from CAFs (Fig. 5A, B). IL-6 has been identified as an important tumor-promoting cytokine that enforces proliferation and anti-apoptotic effects in tumor cells [53]. The expression of IL-6 is markedly elevated in CRC and is closely associated with CRC development [54]. Given that the hallmark of vascular NF-κB activation involves IL-6 production, and it has been demonstrated that the promoter region of the IL-6 gene contains a putative NF-κ B-binding site [55], we investigated IL-6 production in CAF Exo-treated CRC cells. As illustrated in Fig. 5, CRC cells stimulated with CAF-derived exosomes for 48 h exhibited significantly higher IL-6 production compared to CRC cells treated with control PBS or normal fibroblast-derived exosomes (NF Exo). Collectively, our study may provide new insights into the mechanisms through which CAF-derived exosomes exert their oncogenic functions, specifically though the NF-kB p65/IL-6 axis.

## Conclusions

Circulating miRNAs have emerged as promising biomarkers for liquid biopsy, offering potential improvements in the diagnostic capabilities for various human cancers. The present study uncovered the diagnostic potential of a serum-based circulating miRNA signature, comprising miR-20a, miR-122, miR-139, miR-193, and miR-143, as non-invasive biomarkers for CRC. Furthermore, this study demonstrated that exosomal miR-20a-5p originating from CAFs can enhance the proliferation and migration of CRC cells. These effects may be attributed to the down-regulation of PTEN by miR-20a-5p. The study also revealed heightened activity of the NF-κB p65 transcription factor within the nuclei of cells treated with CAF-derived exosomes, leading to increased IL-6 expression. Essentially, this study introduced an explanation in which miR-20a-5p derived from CAF exosomes appears to exert its effects through the miR-20a-5p/PTEN and NF-κB p65/IL-6 axes in CRC cells. Gaining insights into the molecular biology of CAF-secreted exosomal miR-20a-5p holds the potential to enhance our comprehension of CRC progression. Our findings might pave the way for novel therapeutic approaches and the identification of clinical biomarkers for CRC.

### Supplementary Information

**Supplementary Material 1.**

## Data Availability

This article contains Supplementary Information. Full-length blots are presented in Supplementary Figures. The data supporting the findings of this study are available from the corresponding author upon reasonable request.
