# Supplementary material for: Cancer-associated fibroblasts drive colorectal cancer cell progression through exosomal miR-20a-5p-mediated targeting of PTEN and stimulating interleukin-6 production
Source: BMC Cancer. 2024 Apr 1;24:400. doi: 10.1186/s12885-024-12190-0 (PMC10983759; doi:10.1186/s12885-024-12190-0)
Supplement: Supplementary file 1 — Supplementary Material 1. [file 12885_2024_12190_MOESM1_ESM.pdf]

Supplementary Table 1.

Supplementary Table 1. The clinicopathological characteristics of CRC patients

Characteristics	No. of CRC patients (%)	No. of HC individuals (%)
**Age (years)**		
<50	17 (38)	17 (43)
≥50	28 (62)	23 (58)
**Gender**		
Male	35 (78)	30 (75)
Female	10 (22)	10 (25)
**Position**		
Colon	18 (40)	
Rectal	27 (60)	
**Tumor size**		
<2 cm	16 (36)	
≥2 cm	29 (64)	
**LNM**		
Metastasis	19 (42)	
No metastasis	26 (58)	
**Tumor stage**		
I+II	27 (60)	
III	18 (40)	

Abbreviations: CRC, Colorectal cancer; HC, Healthy controls; LNM, Lymph node metastasis

Supplementary Fig. 1

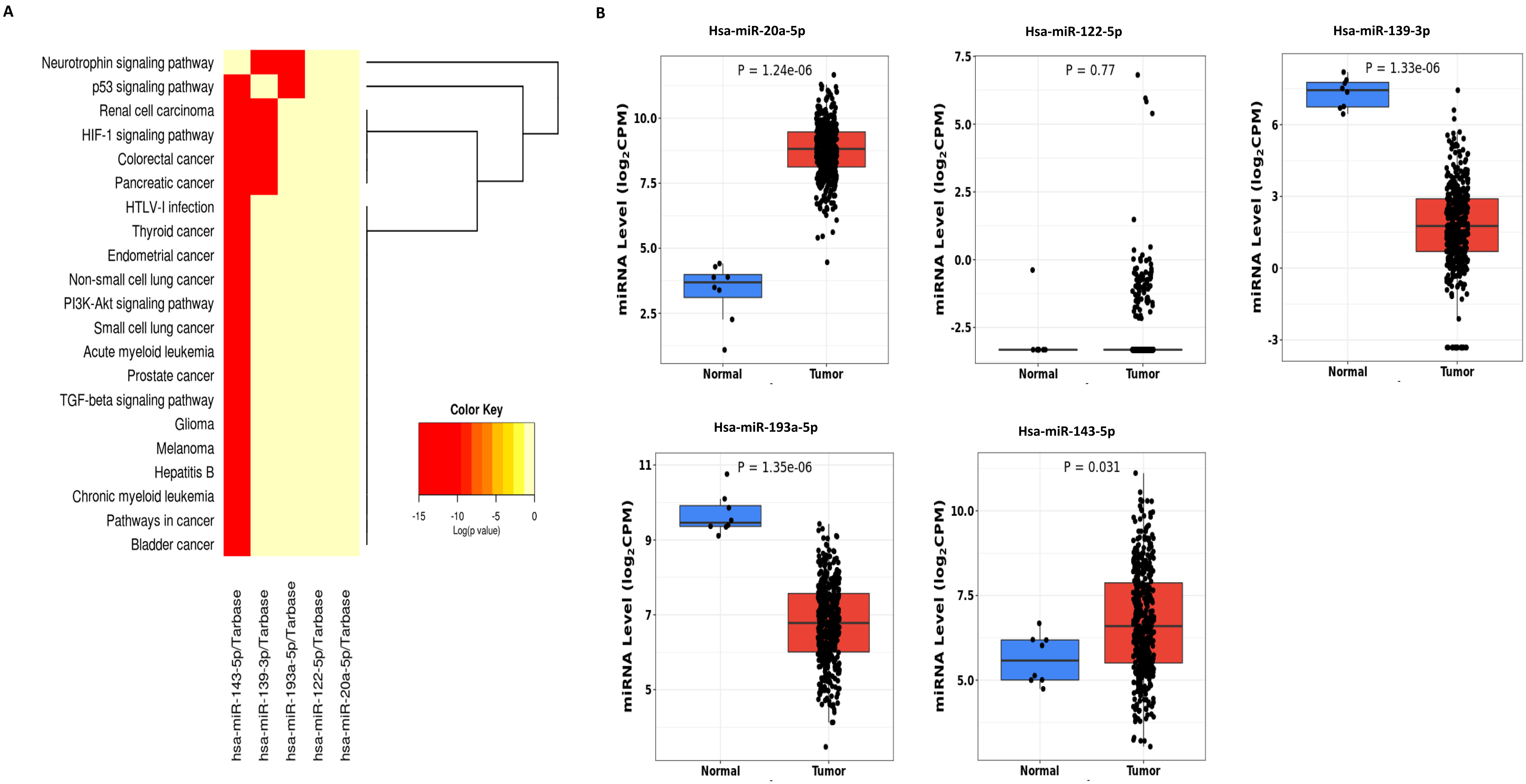

Supplementary Fig. 1. A. DIANA-miRPATH (TarBase) revealed the most critical pathophysiological features associated with each candidate miRNA. miR-20a-5p exhibited the strongest correlation with most of the proposed features. **B.** The expression levels of five candidate miRNAs in CRC patients compared to non-tumor samples. The expression of each miRNA was assessed using CancerMIRNome in CRC.

Supplementary Fig. 2

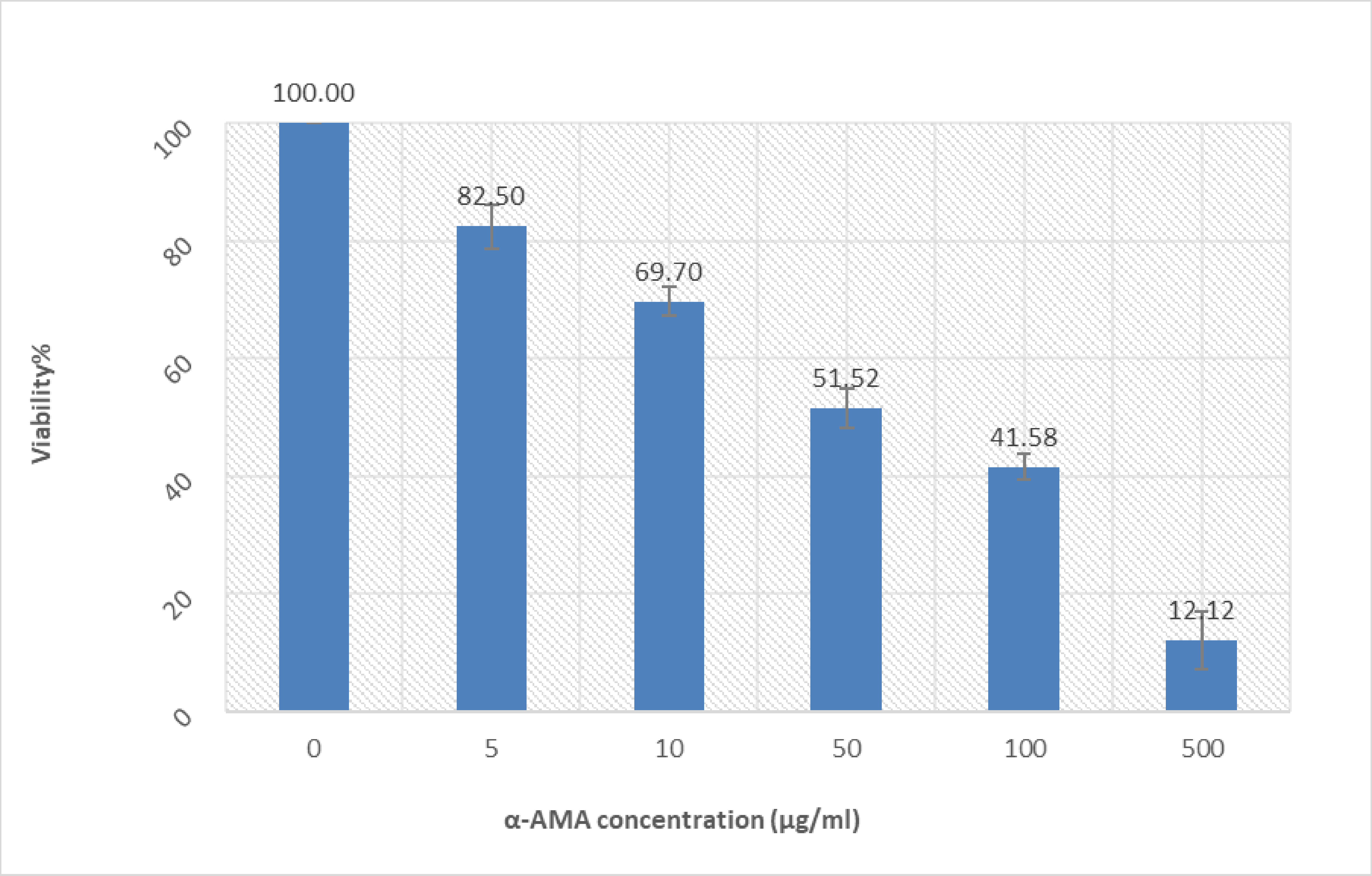

Supplementary Fig. 2. Effects caused by different concentration of α-amanitin (α-AMA) on the viability of SW480 CRC cells. Treatment of SW480 cells with increasing concentrations of α-amanitin (Sigma) for 8 hours demonstrated a dose-dependent reduction in cell survival. These findings were further utilized to determine the IC50 values, which are crucial for subsequent experiments. Notably, at an α-amanitin concentration of 50 μg/mL, the viability of SW480 cells decreased by approximately 50%. Based on these results, the IC50 value of α-amanitin for SW480 cells was established at 50 μg/mL and was subsequently employed in subsequent experimental analyses.

Supplementary Fig. 3

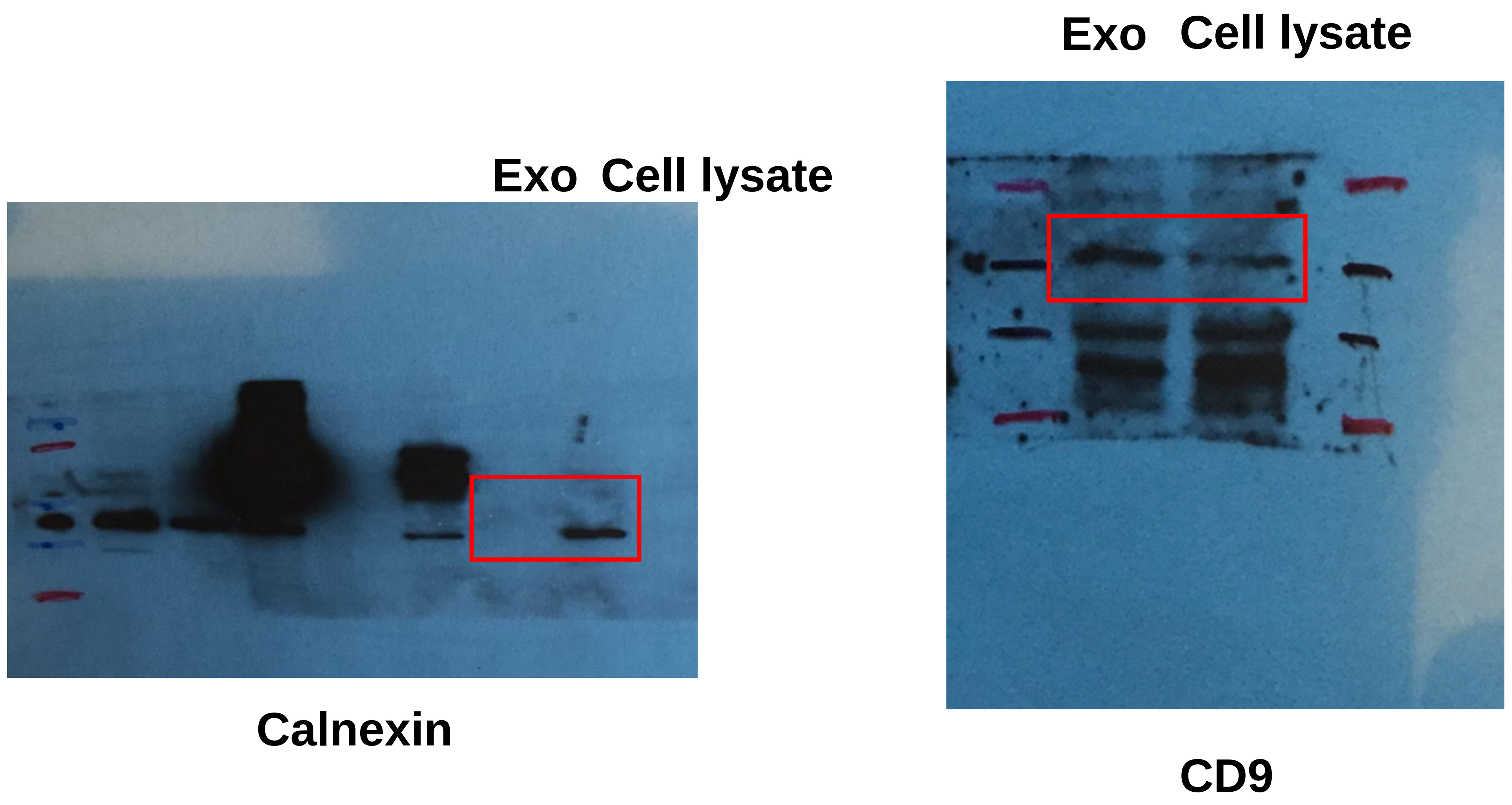

Supplementary Fig. 3. Western blots raw data. The full-length blots of Calnexin and CD9. The same membranes were cut and hybridization with different antibodies according to the molecular weights.

Supplementary Fig. 4

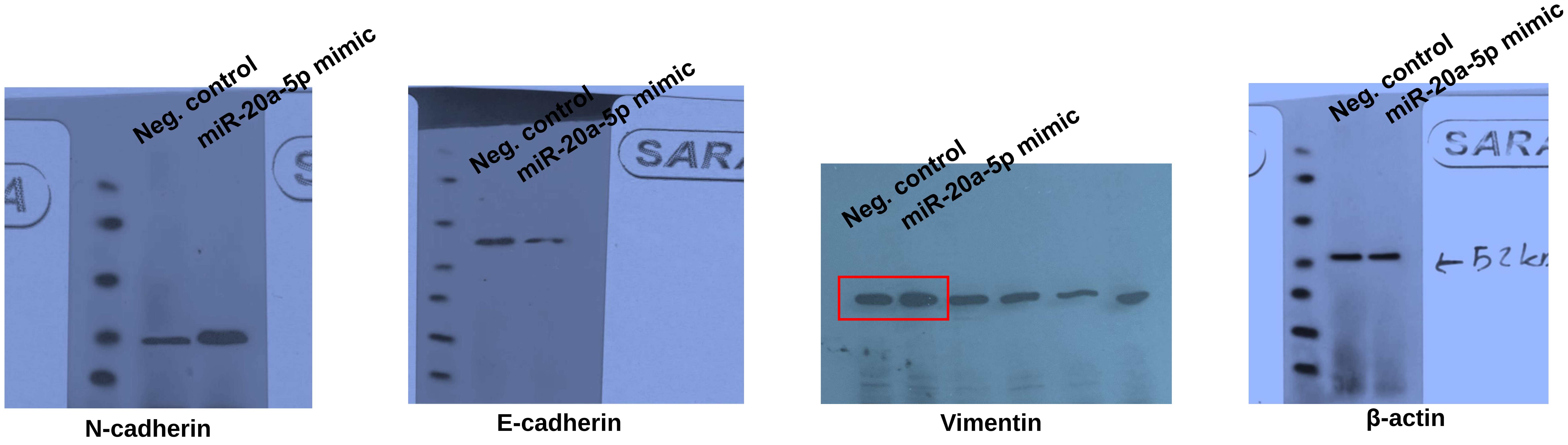

Supplementary Fig. 4. Western blots raw data. The full-length blots of N-cadherin, E-cadherin, Vimentin and β-actin.

Supplementary Fig. 5

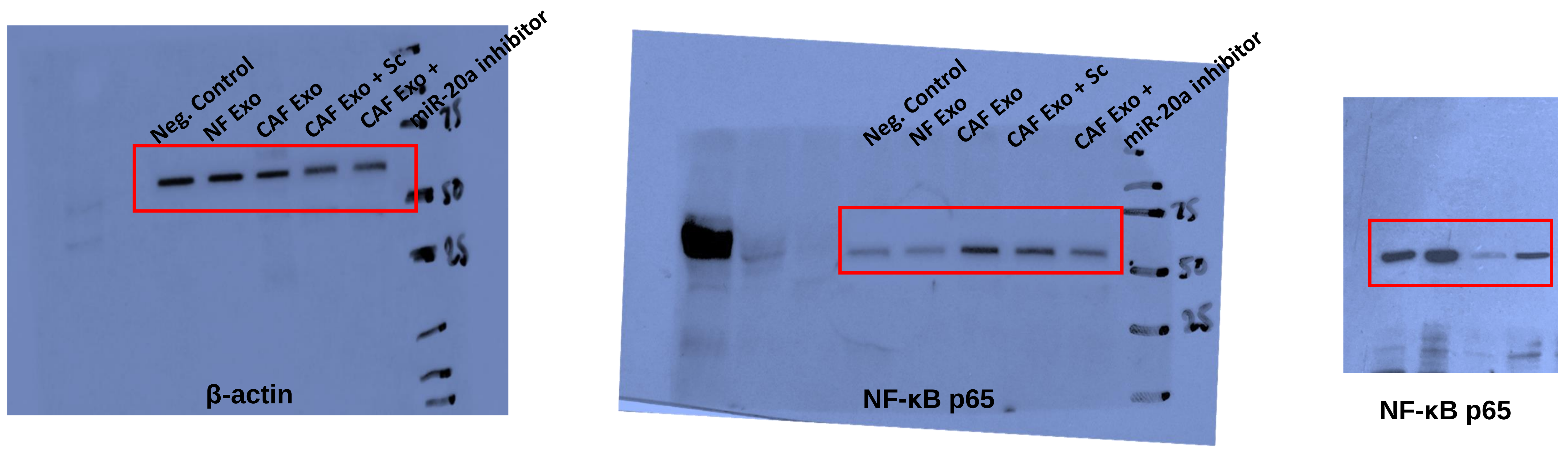

Supplementary Fig. 5. Western blots raw data. The full-length blots of NF-κB p65 and β-actin.
